# CD109 drives pro-tumorigenic cell properties in human non-small cell lung cancer through interaction with desmoglein-2

**DOI:** 10.21203/rs.3.rs-4102385/v1

**Published:** 2024-03-22

**Authors:** Wiebke Lückstädt, Maitreyi Rathod, Lena Möbus, Simon Bub, Ralph Lucius, Felix Elsner, Volker Spindler, Philipp Arnold

**Affiliations:** 1 Anatomical Institute, Kiel University, Germany; 2 Department of Biomedicine, University of Basel, Switzerland; 3 Institute of Anatomy and Experimental Morphology, University Clinic Hamburg-Eppendorf, Hamburg, Germany; 4 Finnish Hub for Development and Validation of Integrated Approaches (FHAIVE,), Faculty of Medicine and Health Technology, Tampere University, 33520, Tampere, Finland; 5 Institute of Pathology, University Hospital Erlangen, Erlangen, Germany.; 6 Institute of Functional and Clinical Anatomy, Friedrich-Alexander-Universität Erlangen-Nürnberg (FAU), Germany

**Keywords:** CD109, desmoglein-2, non-small cell lung cancer, lung adenocarcinoma, cell adhesion, epithelial cell growth

## Abstract

Cluster of differentiation 109 (CD109) is a glycosylphosphatidylinositol (GPI) anchored cell surface protein, expressed on epithelial and endothelial cells, CD4^+^ and CD8^+^ T-cells, and premature lymphocytes. CD109 interacts with different cell surface receptors and thereby modulates intracellular signaling pathways, which ultimately changes cellular functions. One well-studied example is the interaction of CD109 with the TGFβ/TGFβ-receptor complex at the cell surface. CD109 silences intracellular SMAD2/3 signaling and targets TGFβ/TGFβ-receptor to the endosomal/lysosomal compartment. In recent years, CD109 emerged as a tumor marker for different tumor entities and expression of CD109 could be linked to adverse outcome in patients. In this study, we show that silencing of CD109 in human non-small cell lung cancer (NSCLC) cells, returns these cells to an epithelial like growth phenotype. On the transcriptional level, we describe changes in cell-cell contact and epithelial-mesenchymal transition associated gene clusters. At the cell surface, we identify desmoglein-2 (DSG2) as a new interaction partner of CD109 and demonstrate CD109 dependent targeting of DSG2 to the apical cell surface, where it forms desmosomes between apical and basal cell poles. Both, CD109 and DSG2 are genetic risk factors, linked to reduced overall survival in lung adenocarcinoma patients (subtype of NSCLC). In this study, we show the expression of both proteins in the same tumor and suggest a new CD109-DSG2 axis in NSCLC patients that could present a targetable therapeutic option in the future.

## Introduction

**Figure 1:**
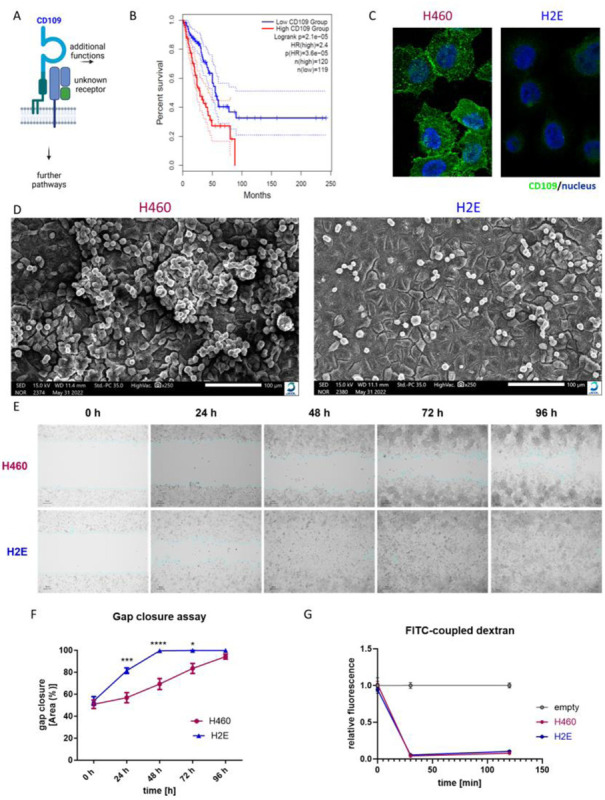
Growth properties of CD109 deficient H460 cells. A) Cartoon of CD109 in complex with a hypothetical cell surface protein and an unknown receptor. B) Kaplan-Meier diagram of the upper and lower quartile of a lung adenocarcinoma cohort derived from *The Cancer Genome Atlas*. The overall survival is shown in dependency of high or low CD109 expression. C) Immunofluorescent image of H460 parental (left) and CD109 deficient cells (right; H2E). D) Scanning electron microscopy image of H460 parental (left) and CD109 deficient cells (right; H2E). E) Gap closure assay experiment showing an exemplary experimental result for H460 parental (upper panel) and CD109 deficient cells (lower panel; H2E). F) Statistical analysis of five independent experiments as shown in E (two-way ANOVA, * indicates p<0.05, *** indicates p<0.001, **** indicates p<0.0001). G) Fluorescein isothiocyanate (FITC)-dextran diffusion assay with H460 (parental) and CD109 deficient cells (H2E) all grown to confluence. Empty wells served as control.

Cluster of differentiation 109 (CD109) belongs to the α2-macroglobulin family of proteins and unlike other members of the protein family, which are soluble, it attaches to the outer membrane leaflet through a glycosylphosphatidylinositol (GPI) anchor (Fig. 1A)^1, 2^. Premature lymphocytes, CD4^+^ and CD8^+^ T-cells, endothelial and epithelial cells express CD109 endogenously^3^. Premature lymphocytes require CD109 to protect themselves from transforming growth factor β (TGFβ) induced maturation^4^. In detail, CD109 interacts with the TGFβ/TGFβ-receptor (TGFβR) complex and suppresses SMAD2/3 phosphorylation^5, 6^. In contrast, CD109 directs the TGFβ/TGFβR complex to the endosomal/lysosomal compartment and induces SMAD7/Smurf2 phosphorylation^7^. At the cell surface, CD109 can be proteolytically processed by e.g. the metalloproteinase meprin β and under non-proteolytic conditions (e.g. absence of a protease), will be increasingly sorted to extracellular vesicles^8^. In recent years, CD109 emerged as a potential diagnostic biomarker in different cancer entities. These include lung cancer^9–11^, breast cancer^12–14^, glioma^15–17^, and ductal pancreatic adenocarcinoma^18, 19^. For some cancer entities, adverse effects for overall patient survival were observed for CD109. As a GPI anchored protein, CD109 does not cross the cell membrane and thus, cannot influence intracellular signaling pathways directly. To modulate signaling cascades, CD109 has to interact with cell surface receptors that have intracellular domains and reside upstream of cellular signaling events. Besides the TGFβ/TGFβR complex, several cell surface receptors emerged as potential interaction partners for CD109 at the cell surface. These include for example the epidermal growth factor receptor (EGFR)^10^ or the cytokine β-receptor gp130^9, 16^ where the EGFR increased AKT-mTOR^10^ and STAT3^20^ induction. Interaction of CD109 with IL-6 and gp130 induced STAT3 phosphorylation and suggested CD109 as an alternative α-receptor for IL-6^16^. However, different signaling pathways linked to increased CD109 expression e.g. YAP/Taz pathway in glioblastoma^15^, still miss detailed information on the cell surface signal transducer. Thus, identification of new cell surface interaction partners and downstream changes in (tumor-) cell behavior are of fundamental scientific and translational clinical interest. In a xenograft mouse model, CD109 expressing tumors showed increased metastases towards liver and lung with a simultaneous increase in gp130 dependent STAT3 phosphorylation^9^. In mice intra-cranially grafted with glioblastoma cells, CD109 positive cells showed higher tumor initiation capacity^15^. In glioblastoma patients, CD109 positivity correlates with reduced overall survival^21^. Metastasis formation and primary tumor growth require different cellular functions such as cell proliferation, migration, loss of cell polarity and cellular reorganization. Although different cell surface interaction partners have been identified for CD109, we are still missing information on cell modulatory effects of CD109 at the molecular level. To understand these different functional properties, new cell surface interaction partners linked to specific functions need to be elucidated (Fig. 1A).

In this study, we utilize a cell model for non-small lung cell carcinoma (NSCLC; H460 cells) and determine differential cell behavior between cells expressing CD109 or those with a CRISPR/Cas9 induced CD109 deletion. Especially differences in cell growth and cell sheet formation/gap closure became evident. We identify proteins of the desmosome adhesion complex as new cell surface interaction partners and demonstrate that CD109 engages with the desmosome protein desmoglein-2 (DSG2) at the cell surface. Deletion of CD109 reduces protein levels of DSG2 in H460 cells and cells return to a rather epithelial phenotype. Both proteins represent risk factors on the transcriptional level for lung adenocarcinoma (LUAD, subtype of NSCLC) patient overall survival. Here, we show the expression of CD109 and DSG2 in the same NSCLC tumor on protein level. Thus, we suggest that CD109 and DSG2 form a targetable protein complex for future translational research.

## Materials and Methods

Unless otherwise stated, chemicals and detergents were purchased from Carl Roth GmbH & Co. KG, Karlsruhe, Germany, and disposable materials from SARSTEDT AG & Co. KG, Nümbrecht, Germany.

### Patient tumor material and immunohistochemistry

The study is covered by an ethical vote of the medical faculty of the University of Erlangen for retrospective translational research activities. After surgical removal of the tumor, tissue was fixed, embedded into paraffin and then sectioned. H&E staining followed routine protocol. For immunohistochemistry, antigens were retrieved through boiling in Tris-EDTA buffer for 10 min. Sections were placed in a wet chamber and washed with TBST (5 min, TBS + 0.1 % Tween 20), incubated with goat normal serum (20 min) and blocked with a blocking kit (Cat# 927301, BioLegend, Inc., San Diego, CA, USA). After washing with TBST (3× 5 min), samples were incubated with primary CD109 (C-9, 1:100) or DSG2 antibody (#610121, 1:200, for details see Table 12) at 4°C overnight. On the next day, samples were washed (3x TBST for 5 min) and incubated with matching HRP-coupled secondary antibodies for 1 h at room temperature. Secondary antibodies were washed off (3x TBST for 5 min), samples were incubated for 1 h with VECTASTAIN^®^ Elite^®^ ABC-HRP Kit (#PK-6100, Vector Laboratories, Inc., Newark, CA, USA), washed again (3x TBST for 5 min) and then incubated with ABC-solution until the desired staining intensity appeared. Samples were washed 3x with ddH_2_O and then hemalaun counter-staining according to Mayer was performed.

### Cell lines and transfection

HEK293T cells were purchased and grown as described before^8^. H460 (NCI-H460) cells were bought at American Type Culture Collection (ATCC, Manassas, VA, USA) and cultured in recommended RPMI-1640 medium with 10 % fetal bovine serum (FBS, PANBiotech, Aidenbach, Germany) at 37 °C, with 5 % CO_2_, and 95 % humidity. Transient and stable transfection of DSG2 into Hek293T cells were performed as previously described^8^. Stable transfected HEK293T CD109 N-term HA-tag (B2) and N-term His-tag cells (A1) have been introduced before^8^.

### Biochemical approaches (cell lysis, SDS-PAGE, and western blot analysis)

Processing the cells for SDS-PAGE and western blot analysis has been performed using standard protocols and described before in detail^8^. Antibodies used for western blot analysis and their dilutions are listed in Table 1.

### Immunoprecipitation

Immunoprecipitation was done either by using Ni-NTA affinity chromatography using ProtinoTM Ni-NTA-Agarose (MACHEREY-NAGEL GmbH & Co. KG, Düren, Germany) as described before^8^ for His-tag purification or by using Dynabeads^™^ Protein G (#10003D, Thermo Fisher Scientific Inc., Waltham, MA, USA) following user manual for HA-tag purification. His-tag purification was performed with N-term. His-tag stable CD109 Hek293T cells (A1), and HA-tag immunoprecipitation with N-term. HA-tag stable CD109 Hek293T cells (B2) as well as N-term. HA-tag stable DSG2 Hek293T cells (C2C). Briefly, cells were lysed in Dulbecco’s phosphate-buffered saline (DPBS, GibcoTM, Carlsbad, CA, United States) + 1 % Triton X-100 and Complete protease inhibitor cocktail without EDTA (Roche, Penzberg, Germany), protein amount was determined using PierceTM BCA Protein Assay Kit (Thermo Fisher Scientific Inc., Waltham, MA, USA)., HA-tag (6E2) antibody 10 μl for 1000 μl lysate was added, and solution was incubated at 4°C over night. Parallel, 30 μl Dynabeads^™^ Protein G per 1000 μg protein were blocked in 2 % BSA solution overnight. The next day, BSA solution was discarded and 1000 μl protein-antibody solution was added to the remaining Dynabeads^™^ Protein G. Samples rolled for 40 min at 4°C. After washing 4x with lysate buffer for 10 min 1x LDS buffer was added, incubated for 5 min and heated for 10 min at 97°C and 600 rpm. Purificated LDS-solution was saved and used for western blot analysis or stored at 80°C for no longer than 3 months.

### Generation of CD109 knock out cells via CRISPR/Cas9

CRISPR/Cas9 knock out cells were created analog to Wöhner et al.^22^. Shortly, material was purchased from Synthego (Gene Knockout Kit v2, Synthego Corporation, Menlo Park, CA, USA). The following guides were used: guide_#1 (GGGCCUGAUGAUCCCUG), guide_#2 (UUCACAGUCACCUGUGA), guide_#3 (AGACUCCUUCUGCUUCC). H460 cells were transfected by electroporation using the Neon^™^ Transfection System (Thermo Fisher Scientific Inc., Waltham, MA, USA) according to manufacturer’s guidelines in duplicates. Single clone selection, DNA purification for further PCR analysis as well as sequencing and protein extraction for western blot analysis have followed. The forward primer 5’ GTTCCTTCTGTGCGGTTCGTG 3’ and reverse primer 5’ CCATCCTCTTCCCCTTCTCCC 3’ have been used for PCR analysis. After gel extraction of PCR products, H460 native cells and CRISPR/Cas9 clones were verified using sequencing primers 5’ TCTGTGCGGTTCGTGGTTTA 3’ and 5’ ATTGGCGCAGTATGGAGTG 3’ for Sanger sequencing perfomed by Azenta (Burlington, MA, USA). Primers were designed and bought from MilliporeSigma (Burlington, MA, USA). Sequencing data was analyzed using SnapGene Viewer Version 7.1.1 (GSL Biotech LLC, Boston, MA, USA).

### mRNA sequencing

H460 parental cells and two CD109 deficient cell clones were grown to confluence in five different experiments. Cells were harvested and total RNA was isolated from the cells using the NucleoSpin RNA Kit (#740955.50, MACHEREY-NAGEL GmbH & Co. KG, Düren, Germany) following the manufacturer’s specifications. Quality control on concentration and integrity of the isolated RNA was performed with the BioTek Epoch, and its Take3 Microvolume Plates, and the software BioTek Gen5 (Version 3.14, Agilent Technologies, Santa Clara, CA, USA) following the manufacturer’s instructions. RNA libraries were prepared for sequencing using the TruSeq Stranded mRNA protocol (Illumina, Inc., San Diego, CA, USA). Post-library quality control included evaluation of library concentrations and fragment sizes (100–400 bp) on a LabChip. All libraries were index barcoded, enabling multiplexed sequencing. All samples were sequenced on the same lane of a flow cell on a NovaSeq 6000 sequencing system (Illumina, Inc., San Diego, CA, USA) with 2×50 bp.

#### Processing of sequencing data:

Illumina standard adapters were trimmed using Cutadapt v3.5 (parameters: --minimum-length 35, --quality-cutoff 20, --overlap 3, adapter: -a AGATCGGAAGAGCACACGTCTGAACTCCAGTCA, -A AGATCGGAAGAGCGTCGTGTAGGGAAAGAGTGT)^23^. Paired reads were mapped to the human reference genome (GRCh38, Ensembl release 108) using Tophat2 v2.1.1 and Bowtie 2 v2.4.5 (parameters: --transcriptome-index, --library-type fr-firststrand --b2-very-sensitive)^24^. Samtools v1.16.1 was used to remove unmapped reads (parameters: view -h -F 4) and to sort the remaining mapped reads (parameters: sort -n)^25^. HTSeq v2.0.2 was used for read summarization (parameters: --order name, --stranded: reverse, --mode union, --minaqual 20) according to the Ensembl release 108 gene annotation (http://ftp.ensembl.org/pub/release-108/gtf/homo_sapiens/Homo_sapiens.GRCh38.108.gtf)^26^.

#### mRNA-Seq analysis:

To identify outliers, that is, samples exceptionally different from the other samples, principal component analysis with transformed sequencing counts (variance stabilizing transformation) were conducted. Differential gene expression analysis was performed with the DESeq2 package (v1.36.0)^27^. Statistical hypothesis testing was performed by the parametric Wald test and subsequent independent filtering of the results. Differentially expressed genes were defined by a false discovery rate (as defined by Benjamini-Hochberg) of <5%. Log fold change estimates were corrected by the DESeq2 inbuilt LFC shrinkage function with the ‘apeglm’ method^28^. To show the top 20 up- and down-regulated genes in a heatmap, it was focused on those genes with a log2 fold change > 1 / < −1 in both clones as compared to the H460 parental cells. Lowly expressed genes (first quartile) and for the remaining genes were removed, calculated an overall log2 fold change by taking the mean of the two log2 fold changes from the pairwise comparison of clone 1 and clone 2 vs. the H460 parental cells, respectively. For the functional enrichment analysis, Clusterprofiler package (v4.4.4)^29^ was used. We performed overrepresentation tests using gene sets from Gene Ontology (GO; http://geneontology.org/) and MSigDb such as the Hallmark pathway set^30^. For the overrepresentation test the differentially expressed genes were used that reached a p-value < 1e-04 in at least one of the clones as compared to the H460 parental cells.

### PCR, rtPCR, agarose gels

DNA extraction was performed using the DNeasy^®^ Blood & Tissue kit (#69504) from QIAGEN (Hilden, Germany) following the DNeasy Blood & Tissue Handbook (07/2020). DNA was measured with BioTek Epoch (Agilent Technologies, Santa Clara, CA, USA). PCR and rtPCR was performed with the forward and backward primer stated above. Standard protocol was used with the GeneAmp^®^ PCR System 9700 (Thermo Fisher Scientific Inc., Waltham, MA, USA). 1 % Agarose gel was used for electrophoresis and monitoring purification and amplification process. GeneRuler 100 bp DNA Ladder (#SM0241; Thermo Fisher Scientific Inc., Waltham, MA, USA) was applied for fragment size control. Quantitative real-time PCR (rtPCR) was done as thoroughly described previously^22^. For DSG2 5′-ATGACGGCTAGGAACACCAC-3′ as forward primer and 5′-GGGTCAGTTTGTGGCTGACT-3′ as reverse primer was utilized^31^. As housekeeping gene GAPDH with fwd 5′-GCACCGTCAAGGCTGAGAAC-3′ and rev 5′-GAGGGATCTCGCTCCTGG-3′ primers have been used^32^. Data was normalized to GAPDH and at least three independent experiments have been performed.

## Immunofluorescence staining

### Verification of CD109 deficient cells after CRISPR/Cas9 approach

200 000 cells per well were seeded in a 12 well-plate with glass coverslips. After 24 h cells were washed, fixed in 4 % PFA solution for 10 min, again washed and incubated with primary antibody CD109 (TEA 2/16) solution prepared in antibody Diluent (#003218, Thermo Fisher Scientific Inc., Waltham, MA, USA) for 60 min in the dark. Afterwards washed with PBS for three times and incubated for 45 min with secondary anti-mouse IgG Alexa Fluor^™^ 488 (#A-21202, Thermo Fisher Scientific Inc., Waltham, MA, USA) in the dark. After washing with PBS again, last staining was prepared with DAPI (#D3571, Thermo Fisher Scientific Inc., Waltham, MA, USA) solution and incubated for 15 min at room temperature (RT). Discarded and washed with ddH_2_O, cells were mounted with Immu-Mount (#9990402, Thermo Fisher Scientific Inc., Waltham, MA, USA). Slides were stored at 4°C until analyzed using Abberior Facility Line multilaser confocal scanning microscope (Abberior, Göttingen, Germany).

### CD109-DSG2 interaction and z-stack experiments

Cells were grown on 13 mm glass coverslips and fixed with ice-cold methanol (MilliporeSigma, Burlington, MA, USA) for 10 min on ice, followed by a blocking step with 3 % BSA and 1 % normal goat serum in 1X PBS for 1 h, in a humidified chamber at RT. Primary antibodies against CD109 (C-9) and DSG2 (#610121) were incubated overnight at 4°C. Secondary antibodies included anti-rabbit IgG Alexa Fluor^™^ 488 (#A-11008, Thermo Fisher Scientific Inc., Waltham, MA, USA) and anti-mouse IgG Alexa Fluor^™^ 568 (#A-11004, Thermo Fisher Scientific Inc., Waltham, MA, USA) were incubated for 1h at RT. DAPI (MilliporeSigma, Burlington, MA, USA) was added for 10min to counterstain nuclei. Cells were washed 3 times with 1X PBS and mounted with ProLong^™^ Diamond Antifade Mountant (#P36970, Thermo Fisher Scientific Inc., Waltham, MA, USA). Image acquisitions were done using Stellaris 8 Falcon confocal microscope (Leica, Wetzlar, Germany) with a HCPL APO CS2 63×/1.40 oil objective, with Z-stacks of 0.2 μm thickness. Image analysis was done with ImageJ software for mean fluorescence intensity measurements.

For primary antibody references and dilutions see Table 2.

### Scanning electron microscopy

Samples were prepared as described previously^33^ with the following changes. Cells were grown to confluence prior to fixation, dehydration and sputtering.

### Transmission electron microscopy

Transmission electron microscopy was performed as described previously^34^ with the following changes. Cells were seeded onto 6-well plastic cell culture dishes. Upon confluence, cells were washed in PBS twice and then fixed with Ito-buffer (2.5% paraformaldehyde, 2.5% glutardialdehyde, 0.1% picric acid in 0.1M cacodylat buffer pH7.3). After embedding samples were cut orthogonally to the cell adhesion surface. Images were acquired on a JEOL1400Plus TEM operating at 120kV acceleration voltage. Magnifications as indicated in the figure legends.

### Gap closure assay

H460 and CRISPR-Cas9 CD109 depleted clones H2E and H1J were seeded, after cell vitality tested over 70 %, to confluency in ibidi culture-Inserts 2–4 well (#81176, #80366, #80466, ibidi GmbH, Gräfelfing-Lochham, Germany). The gap between the cells is independent of the used inserts. Cell counting and vitality measurements have been performed using the TC20 Automated Cell Counter (Bio-Rad Laboratories GmbH, Feldkirchen-Heimstetten, Germany). 5×10^5/ml cells were seeded into the inserts within a 6-well plate and the ibidi manual for wound healing assay was followed. Briefly, after 24 hours the inserts were removed, cells were washed with 1 ml DPBS and additional medium (2 ml) was carefully added. In the migration experiments cells were incubated with 2 ml of medium with 10 μM mitomycin C (#M0503–2MG, MilliporeSigma, Burlington, MA, USA) end concentration for proliferation inhibition. Pictures were taken at 0 h with at least three different coordinates using the Keyence BZ-X800E (KEYENCE DEUTSCHLAND GmbH, Neu-Isenburg, Germany), and repeated at 24 h, 48 h, and 72 h always at the same plate coordinates. The mitomycin C experiments were aborted after 48 h, as the cells started to deteriorate. Original tiff-images were processed with Image J (1.54f, Wayne Rasband and contributors National Institutes of Health, USA) using the plugin of in vitro scratch wound healing assays^35^. Area [%] was taken for further calculations within Microsoft Excel (Microsoft Corporation, Redmond, WA, USA). Mean value of the three technical replicates were taken, and after performing at least three independent experiments, statistical analysis was performed (see below).

### Fluorescein isothiocyanate(FITC)-dextran diffusion assay

H460 parental or CD109 deficient cells were seeded onto 24-well cell culture inserts with a 0.4 μm PET translucent membrane (cellQART) in normal cell culture medium. After cells reached confluence (4 days), medium in the upper chamber was exchanged to a medium that contained 1mg/ml FITC-conjugated dextran (FD-40, MW 40,000; MilliporeSigma, Burlington, MA, USA). After 15 or 60 min cell culture inserts were removed and cell culture medium from the lower chamber was collected and analyzed in a plate reader. As control an empty membrane was used that had no cells seeded on it.

### Proteomics

Sample was delivered in approximately 100 μL of SDS loading buffer. The sample was diluted 1:1 with 1% SDS and 100 mM ammonium bicarbonate (ABC) and the disulfide bonds reduced with TCEP (5 mM) at 65 °C for 30 min, left to cool, and then alkylated with iodoacetamide (12.5 mM) for 30 min at 25°C in the dark. Samples were precipitated onto a mixture of SpeedBeads, Magnetic Carboxylate particles (GE Healthcare, Chicago, IL, USA) using 200 μg of beads. Beads were washed three times with 80% ethanol before being digested overnight with trypsin (300 ng) in 100 mM ABC buffer at 37°C. Supernatant was removed from the beads, dried down and resuspended in LC-MS loading buffer and a portion of the sample was loaded and measured via LC-MS.

### LC/MS measurements

Samples were analyzed on a Dionex Ultimate 3000 nano-UHPLC coupled to a QExactive mass spectrometer (Thermo Scientific, Bremen, Germany). The samples were washed on a trap column (Acclaim Pepmap 100 C18, 5 mm × 300 μm, 5 μm, 100 Å, Dionex) for 4 min with 3% ACN/0.1% TFA at a flow rate of 30 μl/min prior to peptide separation using an Acclaim PepMap 100 C18 analytical column (50 cm × 75 μm, 2 μm, 100 Å, Dionex). A flow rate of 300 nL/min using eluent A (0.05% FA) and eluent B (80% ACN/0.04% FA) was used for gradient separation. Spray voltage applied on a metal-coated PicoTip emitter (10 μm tip size, New Objective, Woburn, MA, USA) was 1.6 kV, with a source temperature of 250°C. Full scan MS spectra were acquired between 300 and 2,000 m/z at a resolution of 70,000 at m/z 400. The ten most intense precursors with charge states greater than 2+ were selected with an isolation window of 2.1 m/z and fragmented by HCD with normalized collision energies of 27 at a resolution of 17,500. Lock mass (445.120025) and dynamic exclusion (30 s) were enabled.

### Database Search

The MS raw files were processed by Proteome Discover 2.2 (Thermo Fisher Scientific Inc., Waltham, MA, USA, version 2.2.0.388) and MS/MS spectra were searched using the Sequest HT algorithm against a database containing common contaminants (45 sequences), the canonical human and bovine database. Enzyme specificity was set to tryptic with three missed cleavages allowed. An MS1 tolerance of 10 ppm and a MS2 tolerance of 0.02 Da was implemented. Oxidation (15.995 Da) of methionine residues was set as a variable modification along while carbamidomethyl (57.02146 Da) on cysteine residues was set as a static modification. Minimal peptide length was set to 6 amino acids and the peptide false discovery rate (FDR) was set to 1%

## Bioinformatics and statistics

### Analysis of The Cancer Genome Atlas (TCGA) data

Data was accessed through the Gepia2 server (http://gepia2.cancer-pku.cn) and Kaplan-Meier diagrams were generated using the *Survival Analysis* tool. Expression of CD109 was assessed using the *Expression DIY* tool. The lung adenocarcinoma cohort (LUAD) was analyzed using the upper and lower quartile (n=120 and 119).

### Protein ontology Term (PoTerm) Analysis

To sort the obtained proteomics data set, we utilized the Panther (www.pantherdb.org). We inserted the protein identifiers and sorted based on the human reference data. We then sorted the proteins according to “Protein Class” and within the class “cell adhesion molecules” obtained the proteins denoted in Figure 3A.

### Z-stack reconstruction and visualization

Z-stacks were transferred into FIJI, color channels were separated and each channel was reconstructed using the “3D Project” tool. Subsequently, reconstructed volumes were converted into .mrc stacks using IMOD’s tif2mrc tool^36^ and transferred to UCSF Chimera^37^ (http://www.cgl.ucsf.edu/chimera) for visualization. Generation of the channel overlap between red and green (co-localization of CD109 and DSG2) was calculated with the UCSF Chimera tool *vop*.

### Statistics

If not differently specified, the results are given as mean ± standard error of the mean (SEM). To determine if two groups of data were significantly different, unpaired non-parametric Mann-Whitney-U rank test was used. Due to small collectives testing for a normal distribution could not be performed. Group analysis were performed with two-way ANOVA followed by post-hoc Sidak’s multiple comparison test. p<0.05 was considered as statistically significant. Statistical analysis was performed by using GraphPad Prism 10.1.2 (GraphPad Software, San Diego, CA, US).

## Results

### Oncogenic potential in patients and depletion of CD109 in H460 cells

Utilizing data from *The Cancer Genome Atlas* (TCGA) reveals a reduced CD109 expression in lung adenocarcinoma (LUAD) compared to the control group (Fig. S1A). However, comparing high and low expressing quartiles from the same cohort shows a significant correlation between reduced overall survival and high CD109 expression (Fig. 1B). For patients within the upper quartile the hazard ratio (HR) is 2.4 times higher than for the low CD109 expressing group (Fig. 1B). In the TCGA list of genes associated with a reduced overall survival in LUAD, CD109 ranges among the top 50 genes when comparing upper and lower quartile (not shown). This finding strongly suggests a pro-tumorigenic role for CD109 in LUAD patients and we utilized H460 cells as a model system to unravel possible CD109 dependent cellular effects. H460 cells present with a partial epithelial character and stem from a male patient’s lung lavage fluid, who suffered from non-small lung cancer^38–41^. In western blot analysis, we identified CD109 expressed as endogenous protein in H460 cells and by means of CRISPR/Cas9 technology deleted CD109 from these cells. From the initial CD109 deficient H460 cell pool, we selected single clones and determined CD109 deficiency by western blot analysis (Fig. S1B). To confirm the deletion on genomic level, we utilized sanger sequencing and found that both clones used for further analysis (H1J and H2E) were hit in Exon 2 of the CD109 gene at nucleotide position 2242 (Fig. S1C). Immunofluorescence images show that CD109 localizes to sites of cell-cell contact in H460 parental cells, whereas the signal is missing in CD109 deficient cells (Fig. 1C). To understand general changes in cell morphology and growth behavior, we seeded cells on glass slides and conducted scanning electron microcopy (Fig. 1D). We found that H460 parental cells grow as an epithelial layer and additionally form three-dimensional cell foci. In contrast, we could not identify these cell foci in CD109 deficient H460 cells (Fig. 1D). Thus, CD109 might play a role for three-dimensional cell growth and attachment. Another hallmark of epithelial cells is their ability to close a gap via the formation of a cellular migration front^42^. A comparison of H460 parental and CD109 deficient cells showed marked differences. While the CD109 deficient cells closed the gap within 48 h, thereby forming a continuous cell sheet, H460 parental cells required significantly longer (96 h) for gap closure (Fig. 1E, F). Additionally, H460 parental cells showed growth in cell foci and migrated rather as individual cells instead of a migratory front into the gap (Fig. 1E, 48 h). To assess the influence of cell proliferation, we conducted the same experiment in the presence of mitomycin C (a proliferation inhibitor). In this experiment, we detected minor differences between H460 parental and CD109 deficient cells, as CD109 deficient cells seem to migrate into the gap a bit better (Fig. S1D, E). However, it appears that upon proliferation, H460 cells attach to each other in a way that allows for the formation of three-dimensional cell growth. This seems not to be the case in CD109 deficient cells. Interestingly, the formation of a tight epithelial barrier is unaffected by the depletion of CD109, as deduced from a trans-well diffusion assay experiment. Here, the same amount of fluorescein isothiocyanate (FITC)-dextran diffused through the H460 parental and CD109 deficient cell layer (Fig. S1F, Fig. 1G). As we identified main differences between H460 parental and CD109 deficient cells in the formation of two-dimensional or three-dimensional cell structures (sheets or foci), we next focused on the origin of these changes. In general, there are two possibilities, first, these changes can occur on the transcriptional level and second, they can result from direct protein-protein interaction.

### CD109 associates with transcriptional changes

**Figure 2:**
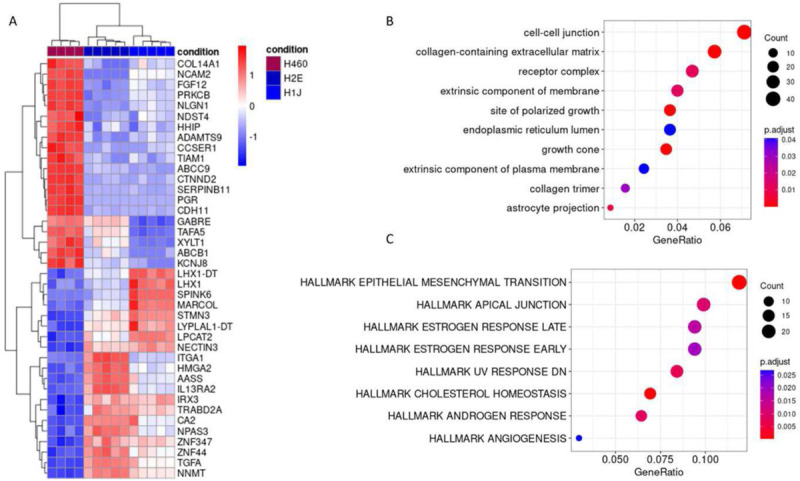
Transcriptomic analysis of H460 parental and CD109 deficient cells. A) Heatmap showing the top 20 up- and down-regulated genes in the CD109 deficient cells based on the average log2 fold change of H2E vs. H460 and H1J vs. H460. The heatmap color code represents row z-scores based on transformed expression counts. B) Enrichment analysis of genes differentially expressed in both CD109 deficient clones as compared to H460 parental cells was performed with Gene Ontology (GO) cellular component terms. All significantly enriched GO terms are shown. C) Enrichment analysis performed with Hallmark pathway gene sets from MSigDb. All significantly enriched pathways are shown. The p-value of the enrichment is indicated by color. The number of differentially expressed genes matching the GO terms and pathways is indicated by dot size.

To analyze CD109 associated changes in gene expression, we conducted mRNA sequencing (RNA-Seq) from H460 parental cells and two CD109 deficient cell clones (H1J and H2E). For this, we prepared five replicates of freshly thawed cell stocks. One of the H460 parental cell experiments was detected as an outlier based on the sequencing data as shown by principal component analysis, and thus we excluded this data set from subsequent analysis (Fig. S2). The other replicates passed our quality control criteria and were included into further analysis. Among the 20 most up- and down-regulated genes, we identified numerous ones connected to cell-cell contacts or extracellular matrix (e.g. Col14A1, ADAMTS9 and ITGA1, Fig. 2A). Enrichment analysis of Gene Ontology (GO) cellular component terms with differentially expressed genes identified several GO terms directly linked to cell-cell contact formation or extracellular matrix (Fig. 2B). Especially the top hit, cell-cell junction is in accordance with our observation of changes in cell-cell contact formation (growth in foci or as sheet). We then tested the differentially expressed genes for enrichment of hallmark pathways from the molecular signature database (MSigDb) and identified “epithelial mesenchymal transition” and “apical junction” as the top two hits (Fig. 2C). Both connect with cancer cell biology. Deduced from the cellular localization site and changes in cell growth behavior between H460 parental and CD109 deficient cells the identified hallmark pathways reflect our cell experimental observations well. Thus, we identify a CD109 dependent change in the transcriptomic signature of H460 cells that associates with cell-cell contact formation, epithelial to mesenchymal transition and extracellular matrix formation. This is a very strong indication that transcriptional changes might contribute to the observed changes in cell growth. To get a full picture, we decided to additionally search for new cell surface interaction partners that could either induce transcriptional changes or directly contribute to cell-cell contact formation.

### Identification of desmoglein-2 as new cell surface interaction partner

**Figure 3:**
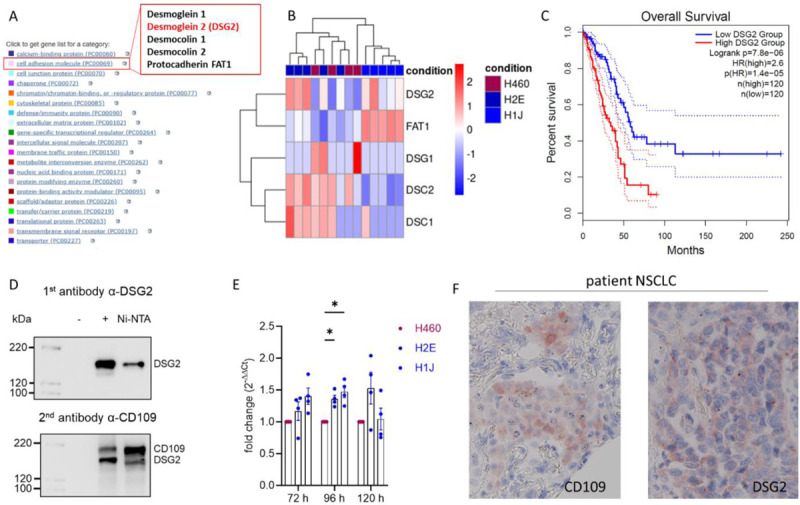
Identification of DSG2 as a new interaction partner *in vitro* and *in vivo*. A) Protein ontology Term (POTerm) analysis of possible interaction partners identified via mass spectrometry fingerprinting (see Tab. S1 for all proteins). Highlighted (insert) a POTerm of cell adhesion molecules that contains four desmosomal cadherins and one protocadherin FAT1. B) Heat map comparing transcriptional values from RNA-Seq data (Fig. 2) for the five cell adhesion molecules identified as potential interaction partners of CD109. C) Kaplan-Meier diagram obtained from *The Cancer Genome Atlas* data showing overall survival in LUAD patients in dependency of DSG2 comparing upper and lower quartile. D) Western blot of a co-immunoprecipitation experiment. Upon precipitation for the His-tag fussed to CD109, endogenous DSG2 was detected as co-precipitated (upper blot). The enrichment of CD109 (successful precipitation) was confirmed with a αCD109 antibody (lower blot). E) rtPCR experiment analyzing DSG2 expression in a time/confluence dependent manner. Expression normalized to H460 control cells and depicted as fold change values (two-way ANOVA * indicates p < 0.05). F) LUAD patient tumor immunohistochemically stained for CD109 (left) and DSG2 (right).

To apply an unbiased approach for the determination of new cell surface interaction partners of CD109, we decided to utilize immunoprecipitation in combination with mass spectrometry protein fingerprinting (Fig. S3A). This method allows us to identify numerous proteins simultaneously, sort them by different properties and then decide on the most promising targets to follow. We expressed N-terminally HA-tagged CD109 in HEK293T cells, isolated the membrane fraction by snap freezing in liquid nitrogen and discarded the cytosol. We lysed the membrane fraction and precipitated with an anti-HA-tag antibody to pull out CD109. Mass spectrometry identified numerous proteins that we co-precipitated (Tab. S1) and we utilized Panther (www.panther.org) to perform protein ontology Term (POTerm) analysis. Among others, we identified a group of cell adhesion molecules (PC00069) that contained desmoglein-1 (DSG1), desmoglein-2 (DSG2), desmocollin-1 (DSC1), desmocollin-2 (DSC2) and the protocadherin FAT1 (Fig. 3A). None of the identified proteins seem to be transcriptionally linked to CD109 as the different genotypes did not form unanimous clusters when we analyzed these five genes in our RNA-Seq data set (Fig. 2B), whereby DSG1 and DSC1 overall showed RNA expression levels close to the detection threshold. Within our proteomics analysis DSG2 showed the highest propensity score and was identified as a potential risk factor in NSCLC/LUAD previously on transcriptional and protein level^43^. In an overall survival analysis based on TCGA data, DSG2 reports a hazard ratio (HR) of 2.6 and the upper quartile associates with significantly lower overall patient survival (Fig. 3C). Thus, we decided to analyze the interaction between DSG2 and CD109 further. To verify the interaction of CD109 and DSG2 with a different method and to exclude interaction through the HA-tag attached to CD109, we utilized differently tagged CD109 and/or DSG2 variants. In a first experiment, we utilized N-terminally His-tagged CD109 and precipitated with Ni^2+^/NTA beads. In the subsequent western blot analysis, we identified DSG2 as co-precipitated and could show the successful enrichment of CD109 (Fig. 3D). In a second experiment, we raised a HEK293T cell line that stably expresses a C-terminally HA-tagged DSG2 (Fig. S3B). We transiently expressed untagged CD109 in these cells and immune-precipitated for the HA-tagged DSG2. As a co-precipitated protein, we identified CD109 (Fig. S3C). Thus, we were convinced that CD109 and DSG2 interact directly and might form an endogenous protein complex. To analyze the transcriptional effect of CD109 deficiency on DSG2 in H460 cells further, we grew H460 parental and CD109 deficient clones for different confluences and harvested them after 72 h, 96 h and 120 h, isolated RNA and performed real-time PCR (rtPCR) measurements. All DSG2 expression values were very similar and only after 96 h we found a small significant increase for DSG2 in CD109 deficient cells when compared to H460 parental ones (Fig. 3E). Thus, we can conclude that CD109 and DSG2 are not transcriptionally linked to a larger extent. In a proof of concept experiment, we utilized tissue sections of a male NSCLC patient of 64 years of age (grade 3, pT2a pN0 L0 V0 pn0) with a tumor size of 35 mm. From H&E stained sections of this resected tumor, we identified the location of the tumor tissue (Fig. S3D) and then utilized immunohistochemistry to stain for CD109 and DSG2. We identified positive staining for both proteins within the tumor (Fig. 3F). To demonstrate specificity of the utilized antibodies, we stained a skin tissue section from a healthy control and identified DSG2 in the basal keratinocyte layer (Fig. S3E)^44^. CD109 staining was most pronounced in the first keratinocyte layers (basal, spinous layer) and we conclude that CD109 and DSG2 might interact directly on the protein level in basal keratinocytes. Next, we analyzed the amount and localization of CD109 and DSG2 on the cell surface to receive possible insight into the mechanism, by which CD109 promotes cell growth in three-dimensional cell foci.

### CD109 stabilizes DSG2 at the cell surface and redistributes it to the apical and basal cell pole

**Figure 4:**
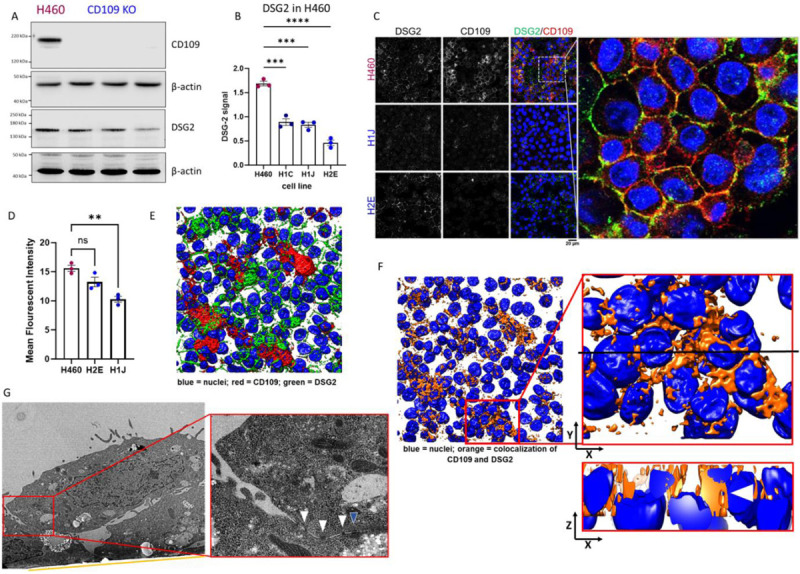
CD109 stabilizes DSG2 at the cell surface and relocates it to apical and basal cell poles. A) Representative western blot of H460 parental and CD109 deficient cells against CD109 and DSG2. B) Statistical analysis of DSG2 band densities normalized against the band density of β-actin from three independent experiments (two-way ANOVA, *** indicates p<0.001, **** indicates p<0.0001). C) Immunofluorescence images of H460 parental and CD109 deficient cells stained against DSG2 (green), CD109 (red) and the nucleus (blue). Insert shows magnified image from H460 parental cells (scale bar = 20 μm). D) Analysis of DSG2 (green) intensity in H460 parental and CD109 deficient cells from three independent experiments (two-way ANOVA, ** indicates p > 0.01). E) Three-dimensional reconstruction of a z-stack image set from H460 parental cells with DSG2 (green), CD109 (red) and the nuclei (blue). F) Three-dimensional reconstruction of a z-stack image set from H460 parental cells where the co-localization of DSG2 and CD109 is displayed in orange and nuclei in blue. Insert (upper) shows view towards the XY-plane and (lower) an image on the ZX-plane. Black line in upper insert indicates sectional view in the lower insert. G) Transmission electron micrograph of H460 cells growing in two layers sectioned perpendicular to the support membrane (orange line). The magnified insert shows cell-cell contacts (white arrowheads) with one contact site showing the ultrastructural hallmarks of a desmosome (blue arrowhead).

To analyze global DSG2 protein levels, we utilized western blot experiments of H460 parental and CD109 deficient cell lines (Fig. 4A). We detected significantly lower endogenous DSG2 protein levels in cells deficient for CD109 when compared with the parental H460 cell line (Fig. 4B). To visualize co-localization of both proteins and analyze the main cell compartment of interaction, we conducted immunofluorescence microscopy (Fig. 4C). It became evident that both proteins meet at the cell surface of a confluent cell sheet (Fig. 4C). When we analyzed the fluorescence signal of DSG2 among H460 parental and CD109 deficient cells, we identified a reduced intensity in CD109 deficient cells (Fig. 4D). Thus, we hypothesize that CD109 and DSG2 meet at the cell surface and that CD109 stabilizes DSG2 there as we detect reduced protein levels upon CD109 deficiency. Additionally, we obtained sectional z-stack images from H460 parental cells, reconstructed them and focused on areas with multiple cell layers (Fig. 4E). To simplify our analysis, we multiplied the two-color channels for CD109 and DSG2 and thus received a single channel that only contains the information where both proteins (CD109 and DSG2) co-localize (Fig. 4F). In a sectional view in the z-x-plane, co-localization of CD109 and DSG2 between apical and basal poles of two cells growing on top of each other becomes evident (Fig. 4F, lower insert with white arrowhead). Thus, we hypothesized that we should identify desmosome-like structures between basal and apical cell poles and utilized ultra-structural histology via transmission electron microscopy to identify such cell-cell contact sites. For this, we embedded H460 cells in resin and sliced them to view along the z-x-plane as in Fig. 4F lower insert. We identified a position with a cell focus, where two cells grow attached to the glass support (Fig. 4G, orange line) and one cell grows on top of these cells without touching the glass support layer (Fig. 4G). In higher magnification, we identified multiple cell-cell contact sites (Fig. 4G, arrows in insert) and one of them showed the typical ultrastructural features of a desmosome contact site (Fig. 4G, blue arrowhead in insert)^34^. Taken together, we identified that DSG2 protein levels decrease in cells deficient for CD109. Additionally, we could show that CD109 and DSG2 co-localize in H460 cells between apical and basal cell poles and that H460 cells form desmosome-like cell contacts between apical and basal sides.

## Discussion

CD109 is a GPI anchored cell surface receptor that interacts with a number of different other cell surface receptors and thereby modulates intracellular signaling properties^5, 7, 9, 10, 16, 45^. Previous reports identified CD109 as an IL-6 receptor independent modulator for gp130 mediated STAT3 phosphorylation^9, 16^, to interact with EGFR directly and thereby change its signaling modalities and expression^10, 11, 46^ and to enhance the Hippo pathway associated signaling transducers Yap/Taz^15^. Among different physiological functions such as protection of stem cells from premature differentiation^4^, detrimental properties have been described mostly linked to tumor biology^47, 48^. For some tumor entities, CD109 was suggested as novel clinical biomarker^19^ and for LUAD patients CD109 was identified among a cluster of risk genes in patients with poor prognosis^49^. In our study, we can now add another layer of complexity to CD109 in tumor biology of NSCLC. We did not only find a transcriptional regulation of genes that associate with cell-cell junction, apical-junctional complex and extracellular matrix, but also describe a direct molecular function that changes cancer cell behavior. We identify DSG2 as a direct cell surface interaction partner of CD109 and show, that deficiency of CD109 returns NSCLC H460 cells to epithelial-like cell sheet formation with regard to growth and migration properties. We show that CD109 and DSG2 are expressed within the same NSCLC tumor, thereby providing proof-of-concept data that both might interact within NSCLC tumors (or a subpopulation of tumors). DSG2 transcription and expression was previously linked to reduced overall survival in LUAD patients^43^. For DSG2, target genes were identified, whose expression level depends on the DGS2 level^50^. However, functional/mechanistic insight remained missing. With CD109, we have now identified a stabilizer for DSG2 at the cell surface. Additionally, we found that both proteins interact in cell foci of H460 cells from the apical to the basal cell pole. In H460 cells deficient for CD109, DSG2 does not locate to the apical site. Our findings are in line with observations from CD109 deficient mice^51^. These mice suffer from a hyperplasia of the basal layer of keratinocytes in the skin^51^. This indicates a decreased cell growth into the third dimension, which might be due to changes in cell-cell contact formation. Interestingly, DSG2 is expressed in the basal keratinocytes as well^44^. Our H460 cell model fits to this observation in mice very well, as we detect largely reduced formation of three-dimensional cell foci after deletion of CD109. We anticipate that CD109 stabilizes DSG2 on the cell surface and targets it from the lateral side to other poles of the cell (apical, basal). Besides CD109, we identified other desmosome proteins within our mass-spectrometry assisted protein fingerprinting approach. We suggest that DSG2 might not be the only cell adhesion protein that is stabilized/relocated in a CD109 dependent manner, but that CD109 stabilizes other cell adhesion proteins as well. CD109 and DSG2 (and eventually the other desmosome proteins) could also form a signaling hub at the cell surface. For both, CD109 and DSG2, a direct interaction with the EGFR was reported^52, 53^ and EGFR signaling contributes to epithelial mesenchymal transition^54, 55^, which is the number one hallmark that we identified in CD109 deficient cells. Interestingly, EGFR expression depends on CD109 in squamous cell carcinoma^46^.

Taken together, we here identify marked differences in cell growth behavior between H460 parental and CD109 deficient cells. RNA-SeqOur transcriptome analysis reveals a genetic association of CD109 with gene clusters associated with cell-cell contact formation, extracellular matrix and epithelial mesenchymal transition. We report a panel of cell adhesion molecules and show a specific interaction of CD109 with DSG2 at the cell surface of H460 cells and expression of both proteins in patient tumor tissue. In H460 cells expressing CD109, we show that desmosomes form between apical and basal cell poles and those cells grow in cellular foci, while CD109-deficient cells show a more epithelial phenotype. In the future, it will be interesting to elucidate whether CD109 stabilizes a protein hub (signaling and/or adhesion) and to identify other protein interaction partners. Additionally, it will be interesting to solve the protein structure of the CD109/DSG2 protein complex to understand the direct molecular interaction interface to design intervention strategies. This could help to directly target this protein complex in NSCLC models in the future.

## Figures and Tables

**Table 1:** List of primary antibodies for Western Blot

Antigen	Host species	Dilution	Retailer
Anti-β-Actin	rabbit	1:5000	Merck KGaA, Darmstadt, Germany
Anti-Human CD109 (TEA 2/16)	mouse	1:1000	BD Biosciences, San Jose, CA, USA
Anti-CD109 (C-9)	mouse	1:1000	Santa Cruz Biotechnology, Inc., Dallas, TX, USA
Anti-DSG2 (F-8)	mouse	1:1000	Santa Cruz Biotechnology, Inc., Dallas, TX, USA
HA-tag (6E2)	mouse	1:1000	Cell Signaling Technology, Inc., Danvers, MA, USA

**Table 2:** List of primary antibodies for immunofluorescence staining

Antigen	Host species	Dilution	Retailer
Anti-Human CD109 (TEA 2/16)	mouse	1:100	BD Biosciences, San Jose, CA, USA
Anti-CD109 (C-9)	mouse	1:500	Santa Cruz Biotechnology, Inc., Dallas, TX, USA
Anti-DSG2 (#610121)	rabbit	1:100	PROGEN Biotechnik GmbH; Heidelberg, Germany

## Data Availability

If not provided in the manuscript or as supplemental material, all data will be shared upon request within the scientific community.

## Research in Context

### Evidence before the study

At the start of our study, CD109 and desmoglein-2 (DSG2) had been independently identified as risk factors in lung adenocarcinoma patients on the transcriptional level. For both, high expression showed correlation with lower overall survival. For DSG2 the same was demonstrated on the protein level in patient tumor sections. For different tumor entities stemming from epithelial cells (e.g. lung- and pancreatic adenocarcinoma and oral squamous cell carcinoma), CD109 had been described as a potential biomarker to correlate with poorer overall patient survival.

### Added value of this study

In our study, we show marked differences in a non-small cell lung cancer (NSCLC) cell line when depleted for CD109 with regard to epithelial growth behavior. We show in RNA-sequencing data that depletion of CD109 correlates with EMT and cell-cell contact formation associated transcriptional signatures. We identified DSG2 as a direct interaction partner of CD109 in an unbiased proteomics approach. Additionally, we show the direct interaction of both proteins at the cell surface and within the same tumor sample. On the mechanistic site, e identify that CD109 directs DSG2 to the apical cell surface and enables cell-cell contact formation from apical to basal, which should not occur in twodimensional epithelia.

### Implications of all the available evidence

In our study, we bring two independent risk factor for NSCLC patients together and show their direct interaction on the protein level. We identify a possible mechanism by which the interaction of both proteins would promote cancer cells growth behavior. The depletion of CD109 largely reduced tumorigenic cell growth behavior. Identifying the direct interaction of CD109 and DSG2 enhances fundamental understanding of tumorigenic cell growth behavior and can additionally be exploited as a targetable protein interaction in NSCLC in future translational research approaches.
